# Rates of Preterm Birth and Low Birth Weight in an Adolescent Obstetric Clinic: Achieving Health Equity Through Trauma-Informed Care

**DOI:** 10.1089/heq.2023.0075

**Published:** 2023-09-13

**Authors:** Amanda N. Noroña-Zhou, Bethany D. Ashby, Georgette Richardson, Amelia Ehmer, Stephen M. Scott, Shaleah Dardar, Ladean Marshall, Ayelet Talmi

**Affiliations:** ^1^ Department of Psychiatry and Behavioral Sciences, Weill Institute for Neurosciences, University of California, San Francisco, San Francisco, California, USA.; ^2^ Department of Pediatrics, University of California, San Francisco, San Francisco, California, USA.; ^3^ Department of Psychiatry, University of Colorado School of Medicine, Aurora, Colorado, USA.; ^4^ Children's Hospital Colorado, Aurora, Colorado, USA.; ^5^ Department of Ob/Gyn, University of Colorado School of Medicine, Aurora, Colorado, USA.; ^6^ Department of Psychological Health and Learning Sciences, College of Education, University of Houston, Houston, Texas, USA.; ^7^ Department of Pediatrics, University of Colorado School of Medicine, Aurora, Colorado, USA.

**Keywords:** birth outcomes, trauma-informed care, racial disparities, integrated behavioral health, adolescent mothers

## Abstract

**Objectives:**

Adolescents who are pregnant and identify as Black are exposed to more societal harms that increase their and their offspring's risk for poor health outcomes. The Colorado Adolescent Maternity Program (CAMP) offers comprehensive, multidisciplinary (medical, behavioral health, nutrition, case management), trauma-informed obstetric care to pregnant adolescents to ensure the healthiest pregnancy and birth possible and pursue health equity. The present study aimed to examine ethnic and racial disparities in preterm birth and low birth weight before and after implementation of a trauma-informed model of care.

**Methods:**

Participants were 847 pregnant adolescents (ages 12–22 years; 41% self-identified as Hispanic, 32% as non-Hispanic Black, 21% as non-Hispanic white) who received prenatal treatment-as-usual (TAU) or trauma-informed treatment. Demographic information, mental health symptoms, and birth outcomes were abstracted from medical records.

**Results:**

Overall, findings provided support that implementation of a trauma-informed model of prenatal care led to equitable birth outcomes across racial and ethnic groups. Specifically, Black adolescents in the TAU group were more than twice as likely to deliver preterm or low birth weight infants compared with white and Hispanic adolescents. In the trauma-informed group, however, there were no statistical differences in birth outcomes across racial/ethnic groups, indicating an elimination of disparities in both preterm birth and low birth weight in this population. These more equitable birth outcomes occurred even in the context of adolescents of color having reported more severe depression symptoms postimplementation.

**Conclusions:**

These findings provide evidence that a health system-level intervention, herein trauma-informed obstetric care for adolescents, can play a meaningful role in the reduction of racial disparities in birth outcomes.

## Introduction

A pregnant person's experiences, including exposure to stress, are directly connected to birth outcomes, infant health, and later child development.^1,2^ With appropriate policies and programs in place within the health system, maternal stress exposure and the effects on child development are preventable and intervenable.^3^ These programs necessitate public health prioritization, considering their potential for long-term, two-generation impacts, and the high prevalence of prenatal stress—about 75% of women experience at least one major psychosocial stressor during pregnancy.^4^ Furthermore, this stress exposure is not equally distributed; marginalized groups, such as communities of color, are disproportionately exposed to harms and stressors that accumulate over the life course and into pregnancy.^5,6^ Particularly, adolescents who are pregnant have been found to have high exposure rates to adversity and trauma,^7,8^ with even higher rates of exposure for Black adolescents.^9^

Thus, obstetric programs utilizing a trauma-informed lens to provide medical and behavioral supports to adolescents who identify as Black (Black adolescents) may be especially important for buffering effects of adversity on maternal and offspring health and development.

Adverse birth outcomes (i.e., preterm birth and low birth weight), one consequence of maternal stress exposure, are common, costly, and developmentally precarious. Approximately 1 in 10 infants in the United States is born preterm (<37 weeks of pregnancy), and about 1 in 12 is born with low birth weight (<2500 g).^10^ While many infants born preterm or with low birth weight develop appropriately, these birth outcomes are associated with increased risk of infant morbidity and mortality,^11–13^ including compromised physical, neurologic, and socioemotional development.^14,15^ Crucially, preterm birth is the *second leading cause of death* among children younger than 5 years.^16^ Infants born preterm or with low birth weight are more susceptible to neuropsychological difficulties in childhood across various domains, including motor function, visuomotor integration, cognitive ability, academic achievement, language, executive function, and behavior problems,^14,17,18^ and there is evidence that these difficulties persist into adulthood.^19–21^

In the United States, annual costs of preterm birth and low birth weight neonatal care are estimated around $5.8 billion—accounting for almost half of all expenditures for infant hospitalizations.^22^ In addition, the aforementioned developmental and health consequences of preterm birth and low birth weight further escalate economic ramifications over the life course.^23,24^ Targeted efforts to reduce rates of these birth outcomes will, therefore, have wide-ranging benefits for individuals, families, communities, and society.

### Disparities in preterm birth and low birth weight

Sociodemographic disparities in preterm birth and low birth weight are well documented. For decades, rates of preterm birth and low birth weight among Black women have been two to three times higher than among white women.^10,25^ In addition, infants born to young mothers (younger than 20 years) are more likely to be preterm or of low birth weight.^26,27^ Intersectional examinations have also revealed that, among adolescent mothers, Black teens have increased odds of preterm birth and low birth weight.^28^ A wide variety of mechanisms underlying these disparities have been examined; converging evidence has highlighted the primary role of racism as a social determinant of these birth outcome inequities.^29^

Racism, defined as a ranking of people into social groups called “races,” which are used to devalue, disempower, and differentially allocate valued societal resources and opportunities to groups defined as inferior,^30,31^ is an insidious stressor that inflicts harm across the life span on a variety of ecological levels, ranging from interpersonal interactions to institutional policies and cultural norms.^32^ The role of institutional racism in health care is long-standing and well established.^32,33^ Historically, the medical field committed many atrocities against Black Americans and, still today, racism in medicine is evident through entrenched health disparities and differential rates of access to health care. Relevant to maternal–child health, Black pregnant individuals are three to four times more likely to experience a complication or death related to childbirth, and Black infants are more than twice as likely as white infants to die in the first year of life.^34,35^

Notably, when Black newborns are cared for by Black physicians, this “mortality penalty” is halved.^36^ In addition, maternal experiences of racial discrimination have been negatively associated with birth weight and gestational length across numerous studies.^5,6,37^ Together, the racist legacy of medicine and persistent health disparities underscore the need for *systems-level change* to achieve equitable outcomes and uphold the ethical principles of our profession.

Navigating the world as a person of color and being chronically exposed to race-related stress and trauma gets “under the skin” and impacts long-term physical and mental health,^38^ starting prenatally. For example, higher frequency of discrimination, as well as a number of contexts in which one has experienced racial discrimination, is associated with increased risk for depression among pregnant women of color.^39,40^ This mental health impact of racism further exacerbates risk, as depression also increases the likelihood of adverse birth outcomes.^41^ Thus, pregnancy represents a sensitive period of development during which a behavioral intervention targeting mental health and adversity, including racial trauma, may confer benefits to both mother and child.

### The Colorado Adolescent Maternity Program

The Colorado Adolescent Maternity Program (CAMP) is an obstetric clinic for pregnant adolescents and young women younger than 22. It is a cooperative endeavor between the Departments of Obstetrics and Gynecology and Pediatrics at a large public medical school in the western United States. Patients are either self-referred or referred from community health providers. Given the specialized focus on pregnant adolescents that is not typically found within general obstetrics, community providers often refer adolescents with psychosocial complexity (e.g., case management needs, low social support) to the clinic. In 2010, CAMP developed a partnership with the medical center's department of psychiatry to address the mental health needs that were identified within the patient population of adolescents who are pregnant. As a result, a trauma-informed, multidisciplinary behavioral health program was established and included positions for psychology and psychiatry providers as well as licensed clinical social workers.

Social workers meet regularly with patients at obstetric visits to build rapport, assess resource needs, and offer psychosocial support. Patients who are identified as having psychiatric concerns are referred to the HEART (Healthy Expectations Adolescent Response Team) program, which includes psychologists who provide diagnostic interviews and psychotherapy and a psychiatrist who provides medication evaluation and management.^42^

The Substance Abuse and Mental Health Services Administration (SAMHSA) provided specific guidance for the provision of behavioral health care with a trauma-informed lens, which aligns with the approach implemented in CAMP.^43^ SAMHSA^14^ defines a trauma-informed approach as follows:

A program, organization, or system that is trauma-informed *realizes* the widespread impact of trauma and understands potential paths for recovery; *recognizes* the signs and symptoms of trauma in clients, families, staff, and others involved with the system; and *responds* by fully integrating knowledge about trauma into policies, procedures, and practices, and seeks to actively *resist re-traumatization* (p. 9).

SAMHSA's six key principles of a trauma-informed approach were used to guide clinic-wide changes. See Ashby et al. for detailed description of the trauma-informed model adopted by CAMP.^42,44^

The relevance of a trauma-informed model of care to an adolescent obstetric clinic is underscored by the intersection of identities in the patient population that increases its exposure to adversities and the subsequent experience of mental health difficulties. As adolescent expectant mothers, they often experience social stigma^45^ and have a higher prevalence of abuse history.^8^ As predominantly adolescents of color, they are exposed to racial trauma in various contexts including health care. Indeed, a previous article documented that more than one-third of CAMP patients disclosed a lifetime history of trauma and abuse.^44^ These accumulated social adversities directly impact maternal mental health and the developing child, and thus, a prenatal trauma-informed intervention has the potential for *intergenerational impact*. For example, Ashby et al.^44^ demonstrated that implementation of the trauma-informed approach described above improved birth weight outcomes as well as prenatal care engagement among CAMP patients.

The present study builds on these findings through examination of whether the new model of care impacted racial disparities in birth outcomes. Such an examination highlights the potential impact of trauma-informed care on populations of pregnant adolescents historically impacted by low birth weight and preterm birth.

### The current study

CAMP provides an ideal framework for studying the impact of a trauma-informed program on racial disparities in birth outcomes among pregnant adolescents. Collection of data from patients preimplementation and postimplementation of the trauma-informed approach to obstetric care affords the comparison of birth outcomes between the two models: prenatal treatment-as-usual (TAU) and trauma informed. Our primary objective was to compare racial disparities in preterm and low birth weight births in the TAU and trauma-informed groups. A secondary objective was to examine mental health symptoms at the time of prenatal care initiation across ethnic/racial groups, to provide some psychosocial context regarding the risk of adverse birth outcomes.

## Method

### Participants

Participants were 847 young women (*M*=18.3 years, range=12–22 years of age) who received obstetric care through CAMP in 2007–2008 (TAU) or 2012–2013 (trauma-informed), and their babies. Of the total sample, 41% identified as Hispanic, 32% Black, 21% non-Hispanic white, and 6% were another racial/ethnic group (e.g., Asian, Native American, mixed race). See Table 1 for a summary of the racial/ethnic composition of groups. All patients' medical expenses were covered by publicly funded health insurance (e.g., Medicaid).

**Table 1.** Ethnic and Racial Composition of the Patients in the Treatment-as-Usual and Trauma-Informed Treatment Groups

Self-identified ethnic/racial group	TAU		Trauma-informed	
	***n***	**%**	***n***	**%**
Hispanic	156	36.7	189	44.8
Non-Hispanic Black	156	36.7	115	27.3
Non-Hispanic white	98	23.1	78	18.5
Total	425	100	422	100

Total includes Asian, Native American, and mixed-race patients, in addition to Hispanic, non-Hispanic Black, and non-Hispanic white patients.

TAU, treatment-as-usual.

### Procedures

All study procedures were approved by the Colorado Multiple Institutional Review Board. Participation in the research study was not required to receive obstetric care from CAMP. Demographic information and birth outcomes of CAMP patients were abstracted from electronic medical records by psychology trainees and faculty members.

### Measures

#### Self-reported race/ethnicity

Patients provided self-report of their race,^*^ selecting one or more of the following categories: American Indian or Alaska Native, Asian, Black or African American, Native Hawaiian or other Pacific Islander, and white. For ethnicity, the patients were asked whether they identify as Hispanic or not Hispanic. Due to low rates of representation for several self-reported racial categories (i.e., American Indian or Alaska Native, Asian, Native Hawaiian, or other Pacific Islander) in the patient population, the categories used in the current sample were Hispanic, non-Hispanic Black, and non-Hispanic white.

#### Birth outcomes

Consistent with the CDC guidelines,^46^ birth weight was recorded in grams, with low birth weight defined as under 2500 g. Gestational age was recorded in weeks, with preterm birth defined as under 37 weeks of gestation.

#### Mental health symptoms

Depression symptoms were measured with the Center for Epidemiologic Studies-Depression scale (CES-D),^50^ a well-validated and widely used 20-item measure of depression symptoms (e.g., sadness, anhedonia, disturbance in sleep and eating). In analyses, we used the established clinical cutoff score of 16 to identify patients with positive depression screeners.^50^

### Data analytic plan

Chi-square tests were used (1) to compare racial/ethnic disparities in rates of low birth weight in the TAU and trauma-informed groups and (2) to characterize mental health symptoms of CAMP's patient population at initiation of prenatal care in both groups (TAU and trauma-informed). Analyses were conducted using SPSS, Version 28. Deidentified data and SPSS syntax are available upon reasonable request.

## Results

### Birth outcomes

Rates of birth outcomes by race/ethnicity are displayed in Table 2. Chi-square tests of the TAU group revealed that Black adolescents had significantly higher rates of preterm birth (14.1% vs. 6.4%, *p*=0.011) and low birth weight (15.5% vs. 7.6%, *p*=0.020) compared with all other racial/ethnic groups. In the trauma-informed group, however, Black patients did not significantly differ in either their rates of preterm birth (8.7% vs. 6.2%, *p*>0.1) or low birth weight (8.3% vs. 6.1%, *p*>0.1) from patients of other racial groups (Table 3). No other chi-square analyses of subgroups revealed significant effects (see Supplementary Tables S1 and S2).

**Table 2.** Birth Outcomes by Race/Ethnicity

	TAU (%)	Trauma-informed (%)
% Preterm birth	9.3	7.0
Hispanic	6.8	6.9
Non-Hispanic Black	14.1	8.7
Non-Hispanic white	6.7	5.1
% Low birth weight	10.9	6.6
Hispanic	8.7	5.9
Non-Hispanic Black	15.5	8.3
Non-Hispanic white	7.1	4.0

Preterm=gestational age <37 weeks, low birth weight=birth weight <2500 g.

**Table 3.** Birth Outcomes for Black Versus Non-Black Patient Populations

	Black (%)	Non-Black (%)	***χ^2^***
**TAU**			
Preterm birth	14.1	6.4	*χ*^2^ (1, *N*=393)=6.48^*^
Low birth weight	15.5	7.6	*χ*^2^ (1, *N*=352)=5.40^*^
**Trauma-informed**			
Preterm birth	8.7	6.2	*χ*^2^ (1, *N*=422)=0.82
Low birth weight	8.3	6.1	*χ*^2^ (1, *N*=405)=0.61

Preterm=gestational age <37 weeks, low birth weight=birth weight <2500 g. ^*^p<0.05.

### Mental health symptoms

Patients in the trauma-informed group (48.3%) were significantly more likely to have positive depression screeners than patients in the TAU group (40.4%; *χ*^2^ [1, *N*=728]=4.46, *p*=0.035). Further examination of the trauma-informed group revealed that Black adolescents (57.5%) were more likely to have positive depression screeners than non-Black adolescents (41.1%; *χ*^2^ [1, *N*=728]=5.30, *p*=0.021), and white adolescents (36.0%) were less likely to have positive depression screeners than non-white adolescents (51.0%; *χ*^2^ [1, *N*=728]=5.56, *p*=0.018). Thus, Black CAMP patients in the trauma-informed group were psychosocially more at risk for adverse birth outcomes. See Table 4 for rates of positive depression screeners across subgroups and time point.

**Table 4.** Positive Depression Screening Rates Across Groups

% Positive depression screener (CES-D)		
Self-identified race/ethnicity	TAU (%)	Trauma-informed (%)
Hispanic	37.7	45.7
Non-Hispanic Black	41.1	57.5
Non-Hispanic white	41.1	36.0

CES-D, Centers for Epidemiological Studies-Depression scale.^50^

## Discussion

Pregnant adolescents who identify as Black are more likely to deliver an infant preterm or with low birth weight compared with other racial and ethnic groups.^28^ These disparities require public health attention considering the prevalence and pronounced sequelae of adverse birth outcomes, including early childhood death.^16^ We examined birth outcome disparities in a sample of racially and ethnically diverse pregnant adolescents who received prenatal care as usual (TAU) or trauma-informed prenatal care in the Colorado Adolescent Maternity Program (CAMP). In the TAU group, established racial disparities in rates of preterm birth and low birth weight in the United States were replicated, such that Black adolescents were *more than twice* as likely to have a preterm or low birth weight baby compared with adolescents of other racial/ethnic groups.

In the trauma-informed group, however, Black adolescents *were not* more likely to have babies with adverse birth outcomes (i.e., preterm birth and low birth weight) compared with adolescents of other racial/ethnic groups. Together, these findings demonstrate that implementation of a health care system-level trauma-informed approach may play a critical role in achieving health equity in adolescent prenatal care. Of note, the observed reduction of racial disparities in the current sample occurred in the context of the Black patient population in the trauma-informed group endorsing higher rates of depression, an established individual-level risk factor for adverse birth outcomes.^41^ This finding suggests that trauma-informed care may serve as a buffer between maternal distress during pregnancy and birth outcomes.

The prenatal period is a particularly sensitive one for not only the child but also the mother^51^; thus, services focused on this developmental period are effective, efficient, and preventative in nature. That the trauma-informed sample had improved racial equity in birth outcomes has notable implications for long-term child and family wellness, as the well-being of mothers and babies is intrinsically interconnected.^52^ Preterm birth is a leading cause of neonatal morbidity and mortality,^53^ and preterm birth and low birth weight increase risk for a wide range of medical complications across the life span.^20^ In addition, having a preterm infant has been linked with poorer family functioning, such as increased debt, financial worry, and parent social isolation.^54^ These burdens are further amplified for families of color.^55^

Considering the larger context of CAMP patients' lives, significant improvements in birth outcomes for Black adolescent mothers likely compound enduring benefits in terms of child health and development and holistic family functioning. Thus, the current findings provide evidence that modifiable health system practices can have a powerful impact on broadly improving the health of communities of color. Such efforts are long overdue.

### Limitations

The current findings should be interpreted in the context of study limitations. Primarily, while the programmatic change in CAMP's model of care afforded examination of birth outcomes before and after integration of a trauma-informed approach, the data cannot speak directly to causing improved birth outcomes. A randomized controlled trial of trauma-informed services within obstetric care would provide needed causal evidence for the effects observed in the present study. Furthermore, our sample lacked adequate representation of additional ethnic/racial groups that have been historically impacted by high rates of preterm birth and low birth weight, such as individuals who identify as Pacific Islander or Native American.^56^ These racial disparities and efforts to dismantle them deserve additional empirical attention.

### Future directions

In addition to the future directions suggested to address limitations of the current study, future research to examine possible mechanisms of change underlying the implementation of trauma-informed care and subsequent improvements in birth outcomes is needed. Mixed-methods approaches utilizing qualitative and quantitative data may be especially informative. For example, interviews with patients and medical providers regarding the trauma-informed approach may identify key mechanisms, such as reductions of racism in the health care system, increased attention to social determinants of health, and increased comfort around addressing mental health concerns. Identification of such mechanisms would support replication of a trauma-informed adolescent model of prenatal care in other health care settings.

To further understand the long-term impacts of a trauma-informed adolescent prenatal clinic and provide additional rationale for policymakers to advocate for such programs, follow-up investigations of the patients and their infants would be valuable. We encourage future studies to examine adherence to well-child health care visits, offspring developmental and health outcomes, and parent–child relationship quality, as the benefits of a trauma-informed approach likely extend far beyond birth outcomes.

## Supplementary Material

Supplemental data

Supplemental data

## Abbreviations Used

CAMP: Colorado Adolescent Maternity Program
SAMHSA: Substance Abuse and Mental Health Services Administration
TAU: treatment-as-usual

## Notes

^*^
The authors acknowledge that race is a political and social construct that often serves as a proxy for the impact of racist practices and structural inequality, not a biological variable, and we designed our analysis with this premise in mind. In addition, categorizations of race and ethnicity are fluid and not universally defined within the United States and therefore definitions are provided to denote participants' self-reported race/ethnicity. In the current study, white is used to refer to people of European ancestry, Black to refer to people of African ancestry, Asian to refer to people of Asian descent, Hispanic to refer to participants from Spanish-speaking countries and/or Latin American descent, and American Indian to refer to participants of indigenous North American descent.^47–49^
