# Supplementary material for: Rates of Preterm Birth and Low Birth Weight in an Adolescent Obstetric Clinic: Achieving Health Equity Through Trauma-Informed Care
Source: Health Equity. 2023 Sep 13;7(1):562–9. doi: 10.1089/heq.2023.0075 (PMC10507928; doi:10.1089/heq.2023.0075)
Supplement: Supplemental data [file Suppl_TableS2.docx]

Supplemental Table 2

*Birth outcomes for White vs. Non-White patient populations*

| Treatment-as-usual | | | |
| --- | --- | --- | --- |
|  | White | Non-White | *x^2^* |
| Preterm birth | 6.7% | 9.9% | *x^2^*(1, *N*=393) = 0.87 |
| Low birth weight | 7.1% | 11.6% | *x^2^*(1, *N*=352) = 1.33 |
| Trauma-informed treatment | | | |
|  | White | Non-White | *x^2^* |
| Preterm birth | 5.1% | 7.3% | *x^2^*(1, *N*=422) = 0.46 |
| Low birth weight | 4.0% | 7.3% | *x^2^*(1, *N*=405) = 0.31 |

*Notes.* None of these chi-square coefficients were statistically different from zero (*p’*s > .05). Preterm = gestational age < 37 weeks, low birth weight = birth weight < 2500 grams.
